# Supplementary material for: Integrated Biological Control Using a Mixture of Two Entomopathogenic Bacteria, Bacillus thuringiensis and Xenorhabdus hominickii, against Spodoptera exigua and Other Congeners
Source: Insects. 2022 Sep 21;13(10):860. doi: 10.3390/insects13100860 (PMC9604179; doi:10.3390/insects13100860)
Supplement: Supplementary file 1 [file insects-13-00860-s001.zip › insects-1901910-supplementary.pdf]

Supplementary Data

Table S1. Primers used in this study

Genes	Orientation	Sequence (5′ - 3′)	Uses	Annealing temperature (°C)	Expected size (bp)
RL32	Forward	ATGCCCAACATTGGTTACGG	RT-qPCR	52.0	240
	Reverse	TTCGTTCTCCTGGCTGCGGA			
Apolipophorin III	Forward	AGTGTCGCCAAGTTGTTCGTG	RT-qPCR	52.0	420
	Reverse	CTCCTGCGCGGTGTTCTGCA			
Attacin 1	Forward	GCTTTCCTCTCCAGGAATATG	RT-qPCR	52.0	276
	Reverse	CCTTAGAGTAAATCCAGTGG			
Attacin 2	Forward	TCCCGAATGTGCCCAACTTC	RT-qPCR	52.0	254
	Reverse	GAAAGATCTGCCGAAAGTAAG			
Defensin	Forward	ATGGGTGTTAAGGTAATAAATGTG	RT-qPCR	52.0	303
	Reverse	GCAACTACATGTATGACTAACGC			
Gallerimycin	Forward	TCAGTCATGAAAGCTTGCGTA	RT-qPCR	52.0	222
	Reverse	TCGCACACATTGGCATCCATTC			
Gloverin	Forward	CGTGGACATCTTCAGGGCC	RT-qPCR	52.0	277
	Reverse	GTCGTGTTCAATGCCACC			
Lysozyme	Forward	ATGCAAAAGCTAACGGTTTTC	RT-qPCR	52.0	385
	Reverse	GATTCTTCCATCCATACCAG			
Transferrin I	Forward	GTCCCTCTCTGTCCTGAAGG	RT-qPCR	52.0	370
	Reverse	CAGAAACACGAAGAAAGATGG			
Transferrin II	Forward	GATGTTCTGGCGCAGCTGTC	RT-qPCR	52.0	288
	Reverse	CCGGCTGAACGCAAACACAG			
Cecropin	Forward	ATCGTTTAGCTTCGTGTTCGC	RT-qPCR	52.0	251
	Reverse	CTTTCTTTTACCACACGGTTG			

Table S2. Control efficacies of different treatments of *B. thuringiensis aizawai* (BtA) and mixtures with the bacterial secondary metabolites of *X. hominickii* against S. exigua infesting Welsh onions in a greenhouse. Survival rates were measured 3 and 7 days after treatment (DAT) to estimate the control efficacies. The experimental units in each treatment were randomly chosen from three blocks.

	Tested insecticide	Before tested insecticide (Insect number/REP)	Live Insect (%) Rep 1	Rep 2	Rep 3	Average	DMRT	Control efficacy (%)
3 DAT	XhE	26.3	88.8	92.5	92.0	91.1	a	3.6
	BtA	31.3	80.6	64.5	78.1	74.4	b	21.3
	BtA+XhE	27.0	48.2	46.4	45.8	46.8	c	50.5
	Control[1]	30.6	96.8	90.9	96.2	94.6	a	-
7 DAT	XhE	26.3	81.4	88.8	84.0	84.7	b	8.4
	BtA	31.3	54.8	51.6	50.0	52.1	c	43.6
	BtA+XhE	27.0	20.6	25.0	20.8	22.1	d	76.1
	Control[1]	30.6	93.7	87.8	96.2	92.5	a	-

[1] Control spray used 5% acetone, which was used to dissolve XhE.
